# Combining Data From Assessments of Leisure, Occupational, Household, and Transportation Physical Activity Among US Adults, NHANES 2011–2016

**DOI:** 10.5888/pcd17.200137

**Published:** 2020-10-01

**Authors:** Geoffrey P. Whitfield, Emily N. Ussery, Susan A. Carlson

**Affiliations:** 1 Division of Nutrition, Physical Activity, and Obesity, National Center for Chronic Disease Prevention and Health Promotion, Centers for Disease Control and Prevention, Atlanta, Georgia

## Abstract

Physical activity occurs in 4 domains (leisure, occupational, household, and transportation), but US surveillance often focuses on leisure-time only. We compared estimates of self-reported leisure-time physical activity and estimates of all-domain activity among adults in the National Health and Nutrition Examination Survey for 2011–2016. During the study period, 38.6% met the aerobic physical activity guideline in leisure-time, 58.5% in leisure-time and occupational/household activity, and 63.7% in all domains. Differences within most subgroups when using all domains were similar to differences when using leisure-time activity only, except that we observed no urban/rural differences in the multidomain assessment. Assessment of multiple domains of activity instead of leisure-time–only activity affects prevalence estimates to a greater extent than it affects subgroup differences.

**Summary**

**What is already known on this topic?**

Assessing all domains of physical activity results in higher prevalence estimates of meeting the aerobic physical activity guideline than assessing leisure-time activity alone, but the implications of multidomain assessment on patterns of participation across subgroups are unknown.

**What is added by this report?**

Compared with leisure-time only assessment, multidomain assessment did not change pairwise comparisons of meeting the aerobic guideline across most subgroups. The lack of urban/rural differences in multidomain assessment was an exception.

**What are the implications for public health practice?**

The choice between comprehensive (multidomain) or brief (leisure-time) physical activity assessment influences prevalence estimates of meeting the aerobic guideline, but it has less influence on differences among demographic and geographic subgroups.

## Objective

Physical activity occurs in 4 domains: leisure, occupational, household, and transportation (1). United States physical activity surveillance often focuses on leisure-time activity (eg, National Health Interview Survey [NHIS] reported in Healthy People 2030 monitoring [2]). Assessing all domains rather than only leisure time may yield higher physical activity estimates (3) and different patterns of participation for demographic and geographic subgroups (4) (eg, if 1 subgroup performs more activity in nonleisure settings than other subgroups). We examined differences in the prevalence of meeting the aerobic component of the *Physical Activity Guidelines for Americans*, second edition (5), using combinations of activity domains, stratified by selected demographic and geographic characteristics.

## Methods

Data were from the 2011–2016 National Health and Nutrition Examination Survey (NHANES), an in-person survey representative of the civilian, noninstitutionalized US population. Participants reported physical activity in 3 sections (combined occupational and household, transportation, and leisure-time domains) using a modified Global Physical Activity Questionnaire (GPAQ), which is publicly available (6) (Table 1). For each prompt, participants who responded yes were asked the number of days per week (frequency) and minutes per day (duration) they performed each activity. We calculated weekly minutes as frequency times duration. Minutes of vigorous-intensity activity were doubled and added to moderate-intensity minutes to calculate moderate-intensity–equivalent minutes (2,5). We classified participants reporting at least 150 moderate-intensity–equivalent minutes per week as meeting the aerobic guideline (5). We used this calculation in 3 combinations of domains: leisure-time activity only (most commonly reported domain); combined leisure-time and occupational/household activity (second most commonly reported domain); and combined leisure-time, occupational/household, and transportation activity (all domains).

**Table 1:** Prompts for Physical Activity Assessment in the National Health and Nutrition Examination Survey, 2011–2016

Order	Domain	Intensity Level	Prompt^a^
1	Not applicable	Not applicable	Next I am going to ask you about the time you spend doing different types of physical activity in a typical week.
2	Combined occupational/household	Vigorous	Think first about the time you spend doing work. Think of work as the things that you have to do such as paid or unpaid work, studying or training, household chores, and yard work. Does your work involve vigorous-intensity activity that causes large increases in breathing or heart rate like carrying or lifting heavy loads, digging or construction work for at least 10 minutes continuously?
3	Combined occupational/household	Moderate	Does your work involve moderate-intensity activity that causes small increases in breathing or heart rate such as brisk walking or carrying light loads for at least 10 minutes continuously?
4	Transportation	Moderate^b^	The next questions exclude the physical activity of work that you have already mentioned. Now I would like to ask you about the usual way you travel to and from places. For example to work, for shopping, to school. In a typical week, do you walk or use a bicycle for at least 10 minutes continuously to get to and from places?
5	Leisure-time	Vigorous	The next questions exclude the work and transportation activities that you have already mentioned. Now I would like to ask you about sports, fitness and recreational activities. In a typical week, do you do any vigorous-intensity sports, fitness, or recreational activities that cause large increases in breathing or heart rate like running or basketball for at least 10 minutes continuously?
6	Leisure-time	Moderate	In a typical week, do you do any moderate-intensity sports, fitness, or recreational activities that cause a small increase in breathing or heart rate such as brisk walking, bicycling, swimming, or golf for at least 10 minutes continuously?

a Only the prompts are presented. When participants answer in the affirmative, follow-up questions assess the usual frequency (days per week) and duration (minutes per day) of participation for each domain/intensity.

b All transportation-related activity is assumed to be of moderate intensity.

Participants self-reported demographic characteristics (sex, age, race/ethnicity, and educational attainment). Urban or rural residence was based on the US Census urban/rural designation provided as a restricted variable (7), as described previously (8). US Census region was determined according to state of residence.

Following all NHANES analytic guidelines, we estimated the prevalence of meeting the aerobic guideline in the 3 combinations of domains, stratified by demographic and geographic characteristics. Within each geographic and demographic stratum, we used adjusted Wald χ^2^ tests with a Bonferroni correction to test for pairwise differences. We included the “non-Hispanic other” subgroup for reference purposes, but we did not include this group in tests or interpretations because of its heterogeneous makeup and small sample sizes. We tested trends across ordered subgroups by using orthogonal polynomial contrasts. Results with a *P* value <.05 were considered significant. We used Stata version 15 (StataCorp LLC) for all statistical analyses. The initial sample included 17,969 adults aged 18 or older; 5.3% were missing at least 1 covariate, and an additional 0.4% were missing physical activity information, resulting in complete data for 94.4% of adults.

## Results

When we considered only leisure-time activity, 38.6% of adults met the aerobic guideline (Table 2). Men were more likely to meet the guideline than women. Adults aged 18 to 24 and 25 to 34 were similarly likely to meet the guideline; the prevalence was lower with older ages. Non-Hispanic White respondents were more likely than non-Hispanic Black and Hispanic respondents to meet the guideline. The prevalence was higher with higher educational attainment, and higher among urban than among rural residents. We found no differences by US Census region.

**Table 2:** Prevalence of Attaining at Least 150 Minutes of Moderate-Intensity^a^ Equivalent Physical Activity Among Adults, National Health and Nutrition Examination Survey, 2011–2016

Characteristic	Leisure-Time Only^b^	Leisure-Time and Occupational/Household^c^	Leisure-Time, Occupational/Household, and Transportation^d^
**Overall**	38.6 (36.7–40.6)	58.5 (57.1–59.8)	63.7 (62.3–65.2)
**Sex**			
Male	42.4 (40.6–44.3)	65.5 (63.9–67.1)	70.7 (69.1–72.3)
Female	35.1 (32.7–37.5)	51.9 (50.2–53.7)	57.3 (55.4–59.2)
**Age group, y**			
18–24	52.0 (48.6–55.5)^e^	71.6 (69.0–74.1)^e^	76.7 (74.2–79.1)^e^
25–34	48.9 (45.7–52.0)^e^	69.7 (67.4–71.8)^e^	75.2 (72.7–77.5)^e^
35–44	39.7 (36.7–42.8)	61.4 (58.8–64.0)	67.1 (64.4–69.7)
45–64	34.3 (31.8–36.8)	55.3 (53.4–57.3)	61.0 (58.9–63.1)
≥65	27.3 (24.9–29.8)	42.4 (39.7–45.1)	46.5 (43.9–49.2)
**Educational attainment**			
<High school graduate	22.0 (20.3–23.8)	46.0 (43.6–48.3)	54.6 (52.3–56.8)
High school graduate	31.9 (29.5–34.4)	56.9 (54.3–59.5)^e^	61.0 (58.7–63.3)
Some college	37.8 (35.8–39.9)	60.4 (58.2–62.5)^e^^,^ f	65.2 (63.1–67.2)
≥College degree	53.2 (50.4–55.9)	64.3 (62.3–66.2)^f^	69.1 (66.7–71.4)
**Race/ethnicity**			
Non-Hispanic White	40.1 (37.8–42.4)	60.4 (58.7–62.1)	64.8 (63.0–66.6)
Non-Hispanic Black	36.0 (33.8–38.2)^e^	54.5 (52.5–56.5)^e^	61.0 (59.1–62.9)^e^
Hispanic	33.7 (31.9–35.6)^e^	55.0 (52.8–57.2)^e^	61.6 (59.4–63.7)^e^
Non-Hispanic other **g**	39.9 (37.4–42.5)	55.2 (52.5–57.8)	63.1 (60.4–65.8)
**Urban/rural residence**			
Urban	40.1 (38.0–42.1)	58.4 (56.8–59.9)^e^	64.3 (62.8–65.9)^e^
Rural	33.4 (30.1–36.9)	58.9 (55.8–61.9)^e^	61.6 (58.2–64.9)^e^
**Census region**			
Northeast	43.0 (39.4–46.6)^e^	59.3 (56.9–61.7)^e^	65.8 (62.7–68.8)^e^
Midwest	36.5 (32.8–40.3)^e^	57.1 (53.5–60.5)^e^	61.7 (58.2–65.1)^e^
South	36.7 (33.5–40.0)^e^	57.5 (55.2–59.8)^e^	62.1 (59.6–64.5)^e^
West	40.3 (36.0–44.6)^e^	60.5 (57.8–63.2)^e^	66.6 (63.7–69.4)^e^

a Minutes of vigorous-intensity activity were doubled and added to moderate-intensity minutes to calculate moderate-intensity–equivalent minutes. Values presented are percentage (95% CI).

b Linear trend for age group (*P* < .001); linear, quadratic, and cubic trends for education (all *P* < .001).

c Linear and quadratic trends for age group (*P* < .001); linear and quadratic trends for education (*P* < .001).

d Linear and quadratic trends for age group (*P* < .001); linear trend for education (*P* < .001).

e,f Within demographic and geographic stratum, estimates that share a letter are not significantly different (Bonferroni-adjusted *P* value for pairwise difference ≥.05). For example, for leisure time and occupational/household physical activity, results for adults with some college were not significantly different from results for high school graduates (they share “e”), nor were they different from results from college graduates (they share “f”).

g For race/ethnicity, non-Hispanic other was not included in pairwise tests.

When we combined leisure-time and occupational/household activity, 58.5% of adults met the aerobic guideline. Pairwise comparisons within most subgroups, including sex, age group, race/ethnicity, and US Census region, were similar to comparisons when we considered only leisure-time activity. As in the leisure-time assessment, prevalence was higher with greater educational attainment, but differences between most and least educated were attenuated, as were differences between some adjacent categories of education. Unlike in the leisure-time assessment, we observed no significant urban/rural differences.

When we considered leisure-time, occupational/household, and transportation activity together, 63.7% of adults met the aerobic guideline. However, pairwise comparisons across most subgroups (sex, age group, race/ethnicity, and region) were similar to comparisons for only leisure-time activity. Differences between the most and least educated were attenuated. Unlike in the leisure-time only assessment, we observed no significant urban/rural differences.

## Discussion

The prevalence of reporting sufficient activity to meet the aerobic guideline was higher when all domains were considered than when leisure-time only was considered; however, pairwise comparisons for most subgroups were similar, with the exception of urban/rural residence. Our findings suggest that assessing multiple physical activity domains rather than leisure-time only affects prevalence estimates more than it affects patterns of differences in subgroups.

Consistent with our findings, other national surveillance systems that focus on leisure-time activity (eg, NHIS) yield lower estimates of meeting the aerobic guideline than the multidomain NHANES assessment (9). Multidomain assessment provides a comprehensive measure, but it requires many questions (eg, 16 in NHANES vs 6 in NHIS), which increases the burden on respondents. The survey space required for multidomain assessment of aerobic activity could also reduce space available for assessing muscle-strengthening activity, which is also important for health (5). Balancing survey length with comprehensiveness is challenging, and our findings illustrate the potential effect of this tradeoff on estimates and subgroup comparisons.

The urban/rural differences observed in leisure-time physical activity were no longer present when occupational/household activity was included, suggesting that rural residents perform more occupational/household activity than urban residents. A previous report showed higher mean minutes of household activity among rural residents than urban residents (4). We extend these findings by assessing leisure-time and occupational/household activity and including estimates of meeting the guideline. Multidomain activity assessments may be needed to accurately monitor urban/rural differences in physical activity participation.

Our study has several limitations. First, self-reported physical activity episodes of 10 minutes or longer may be subject to recall and social desirability biases (10), and associations between reported activity and measured movement may vary by domain (11). Second, our complete-case analysis could bias results if respondents with complete information were different from respondents without. Third, multidomain questionnaires may encourage over-reporting activity because of double reporting in different domains (10). Fourth, leisure-time activity was assessed last in NHANES, which could reduce estimates of meeting the guideline (12). Strengths include a nationally representative sample and analyses in the Research Data Center (7), allowing urban/rural stratification.

Choice of leisure-time–only or multidomain physical activity surveillance requires balancing comprehensiveness with practical concerns, such as survey space and respondent burden. Although the choice of multidomain or leisure-time–only assessment influences estimates of meeting the aerobic guideline, it may have less influence on patterns of differences in demographic and geographic subgroups.
